# Low-Density Lipoprotein Cholesterol Reductions of not Less Than 60 mg/dL Prevent Hemorrhagic Stroke in Hypertensive Populations: A Meta-analysis

**DOI:** 10.31083/RCM36363

**Published:** 2025-05-27

**Authors:** Tao Yan, Lehui Li, Ziying Zhang, Ning Cao, Yuan Xia, Yuan Shen, Haitao Ju, Xingguang Zhang, Nan Zhang

**Affiliations:** ^1^School of Public Health, Inner Mongolia Medical University, 010110 Hohhot, Inner Mongolia, China; ^2^School of Basic Medicine, Inner Mongolia Medical University, 010110 Hohhot, Inner Mongolia, China; ^3^Department of Neurosurgery, The Affiliated Hospital of Inner Mongolia Medical University, 010030 Hohhot, Inner Mongolia, China

**Keywords:** hemorrhagic stroke, LDL-C, hypertension, meta-analysis

## Abstract

**Background:**

The association between low-density lipoprotein cholesterol (LDL-C) levels and the risk of hemorrhagic stroke (HS) detected through different blood pressure statuses remains unclear. Hence, we systematically evaluated the association between LDL-C and HS in populations with and without hypertension.

**Methods:**

We searched PubMed, Cochrane Library, and Embase databases for articles written in English. Only prospective design or randomized controlled trials (RCTs) reporting effect estimates with 95% confidence intervals (CIs) for the relationship between LDL-C and HS were included. We pooled risk ratios (RRs) stratified by blood pressure status and dose–response analyses with a two-stage generalized least squares for trend estimation (GLST) model. Finally, we compared the lower and optimal groups to find the effect of very low LDL-C levels on the risk of HS.

**Results:**

We included seven randomized controlled trials and 9 prospective cohort studies involving 304,763 participants with 2125 (0.70%) HS events. The non-linear trend suggested that LDL-C levels of approximately 80 mg/dL among hypertensive patients and 115 mg/dL among non-hypertensive patients had the lowest risk of HS. Meanwhile, continually lowering LDL-C levels under the optimal (80 mg/dL for hypertensive patients and 115 mg/dL for non- hypertensive patients) LDL-C level would increase the risk of HS in the hypertensive population (RR = 1.84, 95% CI: 1.36–2.50) but not in the non-hypertensive population (RR = 1.15, 95% CI: 0.97–1.36).

**Conclusions:**

The risk of HS can be effectively reduced by controlling LDL-C levels to 60–80 mg/dL in the hypertensive population and 115 mg/dL in the non-hypertensive population. The safety range of controlling LDL-C levels to protect against HS among hypertensive patients is narrower than that among the non-hypertensive population. Additionally, controlling blood pressure might play a positive role in safeguarding against HS by lowering LDL-C levels.

## 1. Introduction

The worldwide global incidence of hemorrhagic stroke (HS) was approximately 3.5 million (42 cases per 100,000 person-years), making it the fourth leading cause of premature death [1]. Numerous studies have shown that lowering low-density lipoprotein cholesterol (LDL-C) levels can reduce the risk of HS [2], but the conclusions remain controversial. A meta-analysis by Masson *et al*. [3] showed no association between LDL-C levels and the risk of HS at levels below 55 mg/dL. In an analysis of Chinese adults, Wu *et al*. [4] found that LDL-C concentrations ≤40 mg/dL were significantly associated with an increased risk of HS. A meta-analysis of 39 clinical trials also showed that lipid-lowering was associated with an increased risk of HS in secondary prevention trials [5]. Recent meta-analyses, including 12 prospective studies [6] and 33 randomized controlled clinical trials (RCTs) [7], have shown that lowering LDL-C levels increases the risk of HS. Thus, the above-mentioned literature shows that the conclusions are not uniform, especially when LDL-C is low. Furthermore, no defined thresholds or clear safety ranges were provided for lipid-lowering to prevent HS.

Hypertension is also an independent risk factor for HS [8]. Patients with hypertension are at higher risk of developing HS compared to individuals with normal blood pressure [9]. It has been suggested that the increased risk of HS may be due to an interaction between high blood pressure and low LDL-C levels [10]. Numerous RCTs have shown that treatment to lower LDL-C and systolic blood pressure (SBP) reduces the risk of HS [11]. Meanwhile, poorly controlled blood pressure and very low levels of LDL-C were shown as the highest rating predictors for stroke [12]. However, few studies have compared the role of LDL-C thresholds in predicting HS risk in normotensive and hypertensive populations. A Scientific Statement from the American Heart Association also highlighted that lipid-lowering therapy does not reduce the risk of hemorrhagic stroke in patients without a history of cerebrovascular disease; however, rational lipid-lowering should be considered by risk stratification [13].

Therefore, this study collected the latest high-quality RCTs and cohort studies to clarify the correlation between different levels of LDL-C and HS risk and further investigate the safe range of LDL-C for protecting HS in hypertensive and non-hypertensive populations. We found that the safety margin for LDL-C control to prevent HS is narrower in hypertensive patients than in non-hypertensive patients. This analysis provides evidence for clinical blood pressure control and safe LDL-C levels.

## 2. Material and Methods

### 2.1 Search Strategy

We searched PubMed, Cochrane Library, and Embase databases for studies examining the association between LDL-C and risk of HS. The following search terms were used: (“hemorrhagic stroke” [MeSH Terms] OR “intracerebral hemorrhage” [Title/Abstract] OR “subarachnoid hemorrhage” [Title/Abstract]) AND (“cholesterol, ldl” [MeSH Terms] OR “low density lipoprotein cholesterol” [Title/Abstract]). The search was limited to studies published before October 2024. The language was restricted to English publications. A detailed search strategy is provided in the **Supplementary Material**. This systematic review has not been registered.

### 2.2 Selection Criteria

Included studies had to meet the following criteria: a prospective design (prospective cohort studies (PCs) or nested prospective case–control study) or RCT; investigate the association between LDL-C level and the risk of HS (intracerebral hemorrhage (ICH), subarachnoid hemorrhage (SAH), or both); report effect estimates (risk ratio (RR), hazard ratio (HR), or odds ratio (OR)) and 95% confidence intervals (CIs) for comparisons between different concentration levels, or sample number and cases in each group to be able to calculate the RR; provide a clear definition of hypertension. Duplicate publications from the same study were excluded. Publications with two or more categories containing zero cases were also excluded.

### 2.3 Data Extraction and Quality Assessment

Two investigators reviewed the included studies and completed standard data extraction forms separately. This form included the following information: author, publication year, study design, study name, location, number of participants, hypertension (%), mean/median age (range), female sex (%), mean/median follow-up duration, endpoint, first occurrence/recurrence, time of LDL-C measurement, details of each LDL-C category such as LDL-C concentration, sample size, cases, effect estimates, 95% CI, and adjusted covariates. The one with the largest number of adjusted variables was extracted for studies reporting several effect estimates.

The quality of the included cohort studies was comprehensively assessed using the Newcastle–Ottawa Scale (NOS) [14]. Studies with more than six stars were regarded as high quality. The quality of the included RCTs was comprehensively assessed using the Cochrane Collaboration’s risk of bias tool, with reference to the Cochrane Handbook [15]. The third investigator resolved disagreements between investigators in data extraction and quality assessment.

### 2.4 Statistical Analysis

Our study regarded RRs as the effect size, with HRs considered equivalent to RRs. For studies reporting results for men and women separately, we combined the estimates using a fixed-effects model to obtain an overall RR of HS for an individual study. A random-effects model was used with *I*^2^ > 50% [15] or *p*_heterogeneity_ < 0.05; otherwise, a fixed-effects model was used. We first calculated the pooled RRs and 95% CIs for median versus low levels of LDL-C. We defined the median group as follows: if the trend in RRs was a line with groups of LDL-C concentration, the middle LDL-C concentration group was considered the median group; if the trend in RRs was a curve, the group with the minimum RR was considered the median group. The low LDL-C concentration group, also defined as the reference group, was the lowest LDL-C concentration group in each study. Subgroup analysis was stratified by reference groups, age, proportion of sex, and follow-up time to find other potential factors that affect the relationship between LDL-C concentration and the risk of HS. Then, we focused on whether there was a difference in RRs among populations with and without hypertension. Hypertension was defined as SBP ≥140 mm Hg and/or diastolic blood pressure (DBP) ≥90 mm Hg or current antihypertensive treatment. The hypertension group was defined as the proportion of hypertensive participants over 60%, while the non-hypertension group was defined as the proportion of hypertensive participants less than 40%. When we pooled the RRs, we also conducted a sensitivity analysis in which we calculated pooled RRs for studies with proportions of hypertensive participants over 60%, 65%, and 75%, respectively. Meta-regression was also conducted to eliminate the effect of potential factors on the relationship between LDL-C concentration and risk of HS, such as age, sex, LDL-C concentration of reference group, and follow-up time. We then investigated the shape of the relationship between LDL-C and HS in a dose-response analysis using a two-stage generalized least squares model for trend estimation (GLST) model, using restricted cubic splines with three knots at flexible percentiles [16, 17] to obtain an optimal fitted curve. For each study, we extracted the mean or median of the categories of LDL-C. If neither the mean nor the median was reported, we calculated the midpoint of each category. For studies with an open-ended highest or lowest LDL-C category, we assumed that the interval was the same as that of the nearest category. We also extracted the number of cases, sample numbers, RRs, and 95% CIs for each category of LDL-C [18]. If the reference category was not the lowest LDL-C, we used the method described by Orsini to translate it to the lowest group [19]. If the unit of LDL-C was mmol/L, the unit was converted to mg/dL by multiplying by 38.67 [20, 21]. Considering studies using different lowest LDL-C concentrations as reference are unsuitable for the GLST model [22], we divided the original studies into three groups according to the LDL-C concentration of the reference group <50 mg/dL, <70 mg/dL, and <100 mg/dL. Finally, we defined the group included LDL-C level with the lowest pooled RR as the optimal group and compared the lower group adjacent to the optimal group and the optimal group to find the effect of very low LDL-C level on the risk of HS.

Heterogeneity was mainly assessed using the *I*^2^ statistic. We considered low, moderate, and high *I*^2^ values of more than 25%, 50%, and 75%, respectively [23]. Potential publication bias was visualized using a funnel plot and estimated using Egger’s and Begg’s tests. Sensitivity analysis was performed by removing one study at a time and then evaluating the remaining studies. All statistical analyses were performed with Stata 16.0 (StataCorp LLC, College Station, TX, USA). A threshold of *p* < 0.1 was used to determine whether heterogeneity or publication bias was present [15]. Otherwise, *p* values were two-sided, with a significance level of 0.05.

## 3. Results

### 3.1 Literature Search

**Fig. 1.**
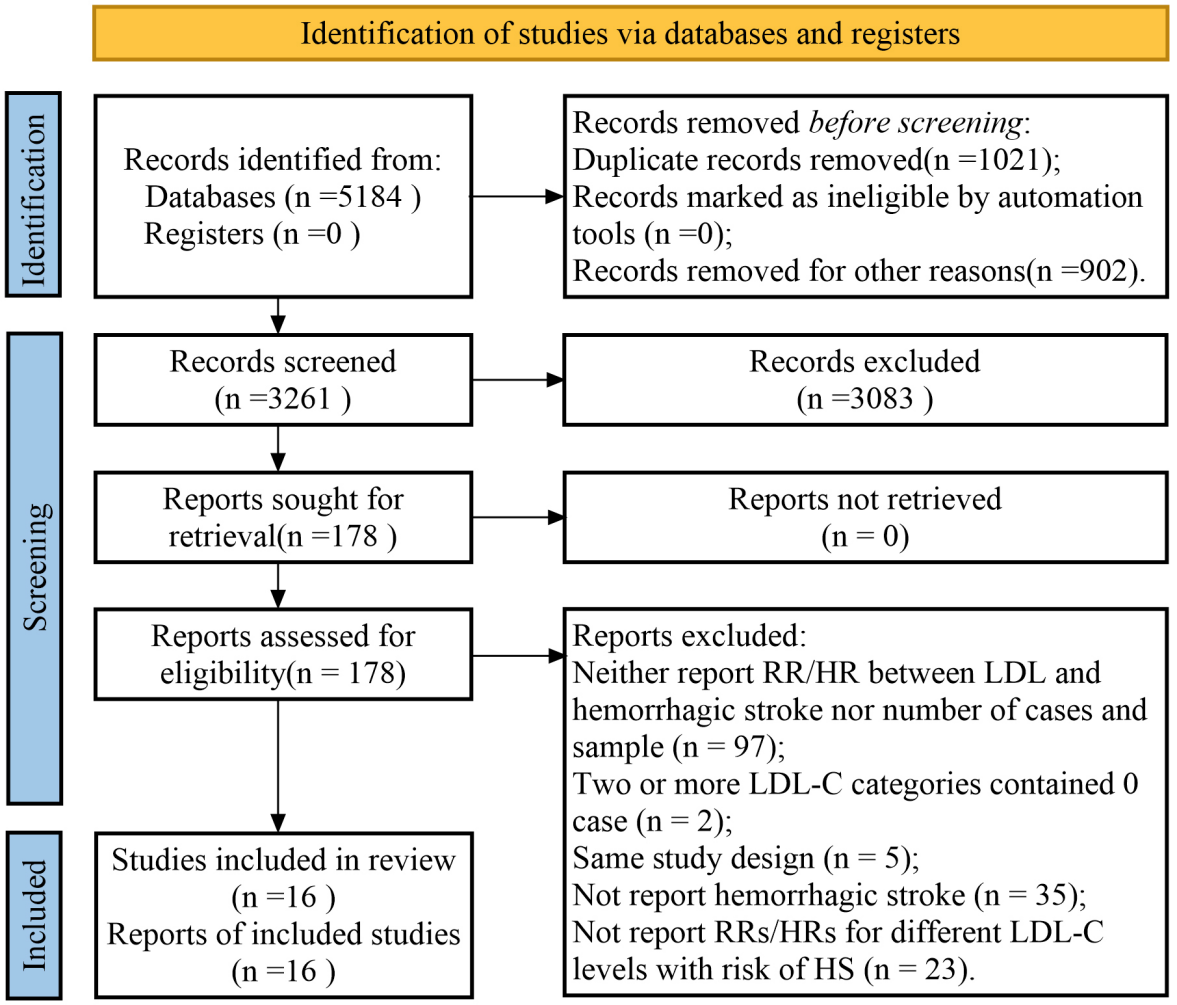
**A flowchart of the selection process in the study**. RR, risk ratio; HR, hazard ratio; LDL-C, lowering low-density lipoprotein cholesterol; LDL, lowering low-density lipoprotein.

Fig. 1 shows the results of the study selection process. The initial search yielded 1574 studies from PubMed, 1840 from the Cochrane Library, and 1770 from Embase. After excluding duplicates, non-original articles, and irrelevant studies, 178 potentially eligible studies were screened. We excluded studies without information on the studied variables and ultimately included 16 studies in the final meta-analysis [9, 24, 25, 26, 27, 28, 29, 30, 31, 32, 33, 34, 35, 36, 37, 38]. A manual search of the reference lists of these studies did not yield any new eligible studies.

### 3.2 Study Characteristics

Sixteen studies were included, with 7 RCTs [25, 26, 27, 29, 31, 35, 37] and 9 PCs [9, 24, 28, 30, 32, 33, 34, 36, 38] (**Supplementary Table 1**), involving 304,763 participants with 2125 (0.70%) HS events. Nine studies ascertained HS as the endpoint [26, 27, 29, 31, 32, 33, 35, 36, 38], and seven studies ascertained ICH as the endpoint [9, 24, 25, 29, 33, 36, 38]. Seven studies were conducted in Asia [9, 24, 28, 29, 30, 34, 38], two in Europe [32, 33], three in North America [26, 36, 37], and four in countries on more than two continents [25, 27, 31, 35]. One study only included women [26], whereas all others included men and women. Only one study [37] focused on recurrence; the remaining studies defined the first occurrence as the outcome. Twelve studies [9, 24, 26, 28, 29, 30, 32, 33, 34, 35, 36, 38] estimated the relationship between HS and LDL-C measured at baseline; four studies [25, 27, 31, 37] used the LDL-C value after taking lipid-lowering medicine. Seven studies [25, 28, 29, 30, 31, 33, 35] reported RRs among hypertensive participants or the study population comprising more than 60% of participants with hypertension. Six studies [9, 24, 30, 32, 34, 35] adjusted the RRs with at least one other type of lipid, including high-density lipoprotein (HDL), total cholesterol, or triglyceride (TG).

### 3.3 Quality Assessment Results

PCs were assessed using the NOS (**Supplementary Table 2**), and RCTs were evaluated via the Cochrane Collaboration’s risk of bias tool (**Supplementary Fig. 1**). The scores of the nine PCs were seven or more; thus, all studies were considered high quality. For the RCTs, 100% had a low risk of reporting bias, and 71% had a low risk of attrition bias, which are important risks for our analysis. Furthermore, 85% of the RCTs had a low risk of performance bias and detection bias, and most RCTs had an unclear risk of selection bias and other biases.

### 3.4 Relationship Between LDL-C and HS

**Fig. 2.**
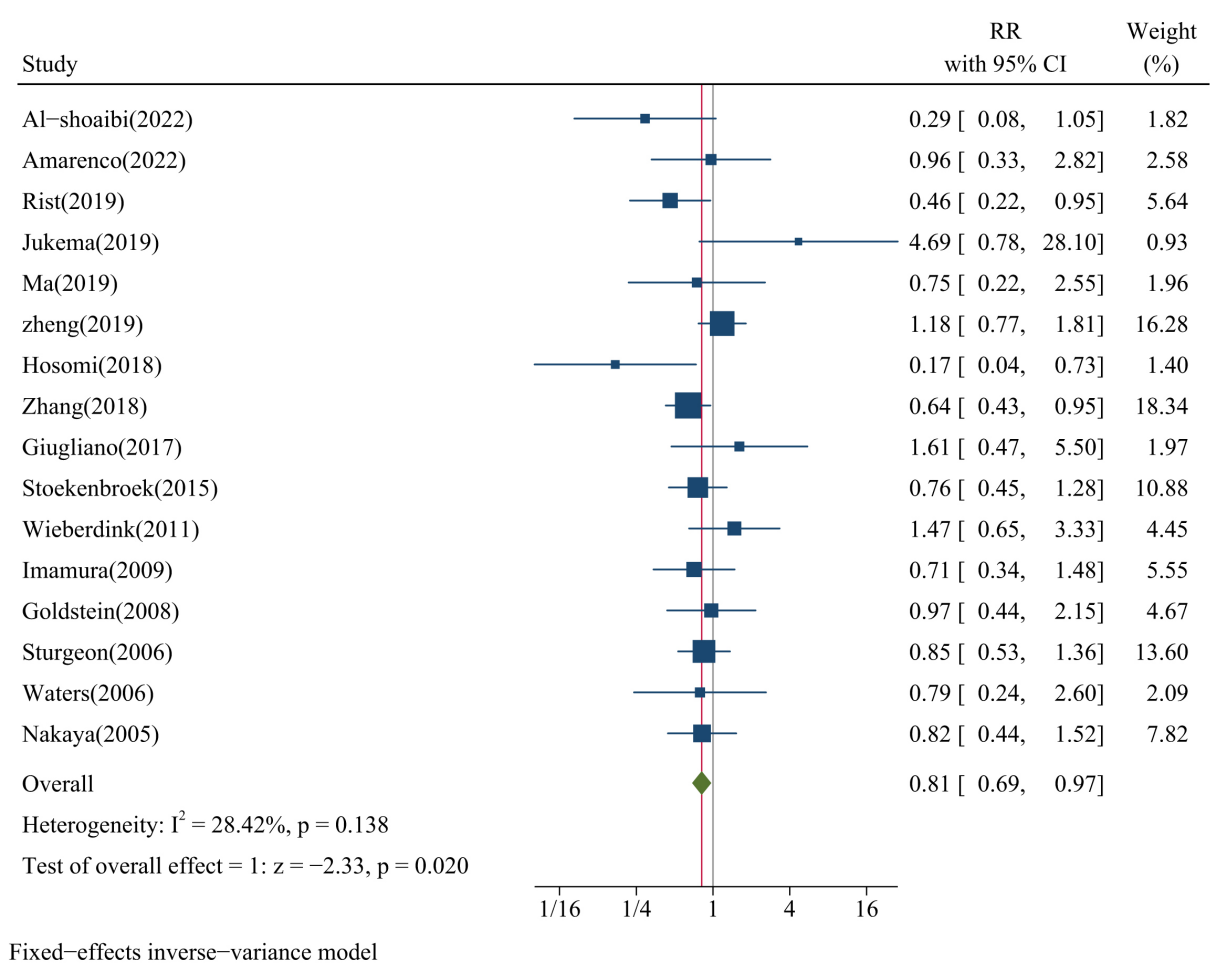
**Forest plots of LDL-C and risk of HS**. CI, confidence interval; IV, inverse variance method.

We pooled 16 studies that provided the RR between categories of LDL-C levels (median versus low) and the risk of HS and found a significant relationship between LDL-C and the risk of HS (pooled RR = 0.81, 95% CI: 0.69–0.97, *p* = 0.020; Fig. 2), with low heterogeneity (*I*^2^ = 28.4%, *p* = 0.138; Fig. 2). The funnel plot (**Supplementary Fig. 2**), Egger’s test (*p* = 0.938), and Begg’s test (*p* = 0.893) showed no significant publication bias. The sensitivity analysis results suggested that the pooled RR was not influenced by any single study (RR range: 0.76–0.86; **Supplementary Fig. 3**).

**Fig. 3.**
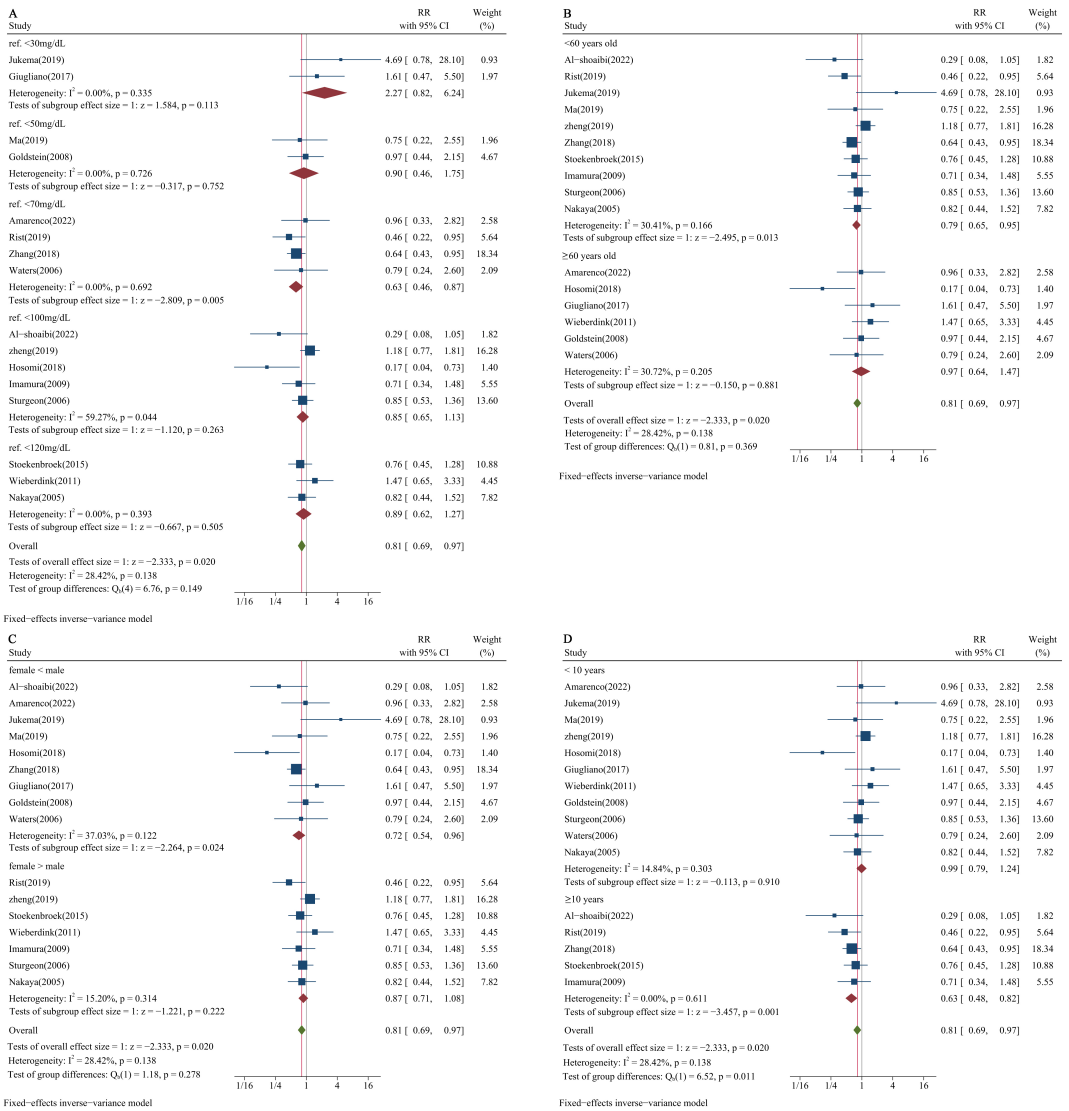
**Subgroup analysis of LDL-C concentration and risk of HS**. (A) Reference groups of LDL-C concentration stratified subgroup analysis. (B) Subgroup analysis stratified by age. (C) Subgroup analysis stratified by proportion of sex. (D) Subgroup analysis stratified by follow-up time.

We also conducted subgroup analysis stratified by reference groups, age, proportion of sex, and follow-up time to find other potential factors affecting the relationship between LDL-C concentration and the risk of HS. The results presented a significant relationship between LDL-C concentration and risk of HS when the reference group was below 70 mg/dL (RR = 0.63, 95% CI: 0.46–0.87, *p* = 0.005; Fig. 3A), when age was below 60 years old (RR = 0.79, 95% CI: 0.65–0.95, *p* = 0.013; Fig. 3B), when the proportion of females was less than males (RR = 0.72, 95% CI: 0.54–0.96, *p* = 0.024; Fig. 3C), and when follow-up time was more than 10 years (RR = 0.63, 95% CI: 0.48–0.82, *p* = 0.001; Fig. 3D).

### 3.5 Relationship Between LDL-C and HS among Hypertensive and Non-Hypertensive Populations

**Fig. 4.**
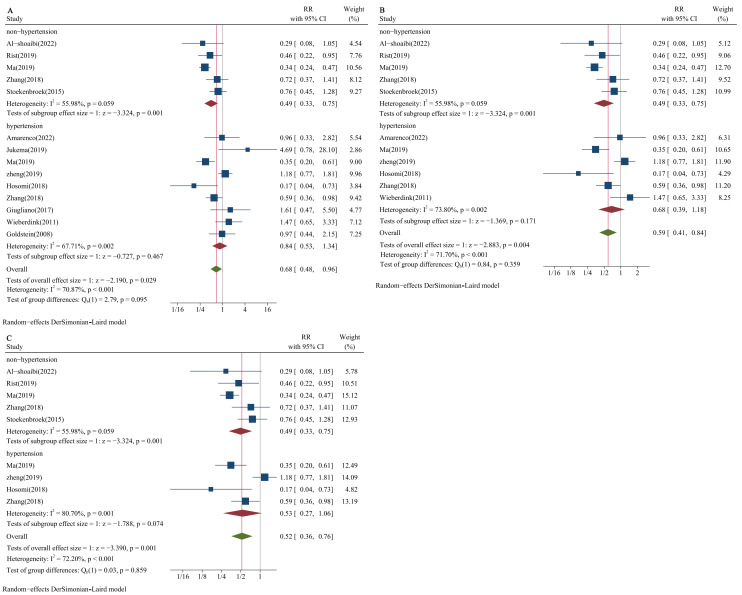
**Forest plots of LDL-C and risk of HS stratified by blood pressure status**. (A) Proportion of hypertension: 60%. (B) Proportion of hypertension: 65%. (C) Proportion of hypertension: 75%. DL, DerSimonian-Laird.

The results for proportions of the study population with hypertension of 60%, 65%, and 75% were consistent. The pooled RRs for the risk of HS between the median and low LDL-C level groups in the hypertensive population were 0.84 (95% CI: 0.53–1.34; Fig. 4A), 0.68 (95% CI: 0.39–1.18; Fig. 4B), and 0.53 (95% CI: 0.27–1.06; Fig. 4C) for the above three proportions, respectively; the result of pooled RR among non-hypertensive populations was 0.49 (95% CI: 0.33–0.75; Fig. 4A–C).

We also conducted a meta-regression analysis to eliminate the effect of potential factors on the relationship between LDL-C concentration and the risk of HS. The results showed that hypertension remained an independent factor affecting this relationship (RR = 1.486, 95% CI: 1.039–2.216, *p* = 0.033; Table 1).

**Table 1.** **Meta-regression of LDL-C and risk of HS**.

Variate	RR	95% CI for RR		*t*	*p*-value
		Low	Up		
Hypertension	1.486	1.039	2.216	2.39	0.033
Follow-up time	1.014	0.572	1.796	0.05	0.959
Proportion of sex	1.565	0.697	3.512	1.20	0.253
Reference group of LDL-C	0.847	0.585	1.227	–0.97	0.351

HS, hemorrhagic stroke.

### 3.6 Dose-response Analysis Between LDL-C and HS among Hypertensive and Non-Hypertensive Populations

To obtain actual, precise dose-response relationships, we divided studies with hypertensive populations with reference LDL-C levels in the original studies into three groups: <50 mg/dL, <70 mg/dL, and <100 mg/dL, respectively. We detected a significant non-linear relationship and linear relationship (except reference group <100 mg/dL) between LDL-C and HS among the hypertensive populations of the above three groups (*p* for non-linear relationship: 0.016, 0.041, 0.002; *p* for linear relationship: 0.005, 0.019, 0.854, respectively) and for non-hypertensive populations (*p* for non-linear relationship <0.0001; *p* for linear relationship 0.019).

**Fig. 5.**
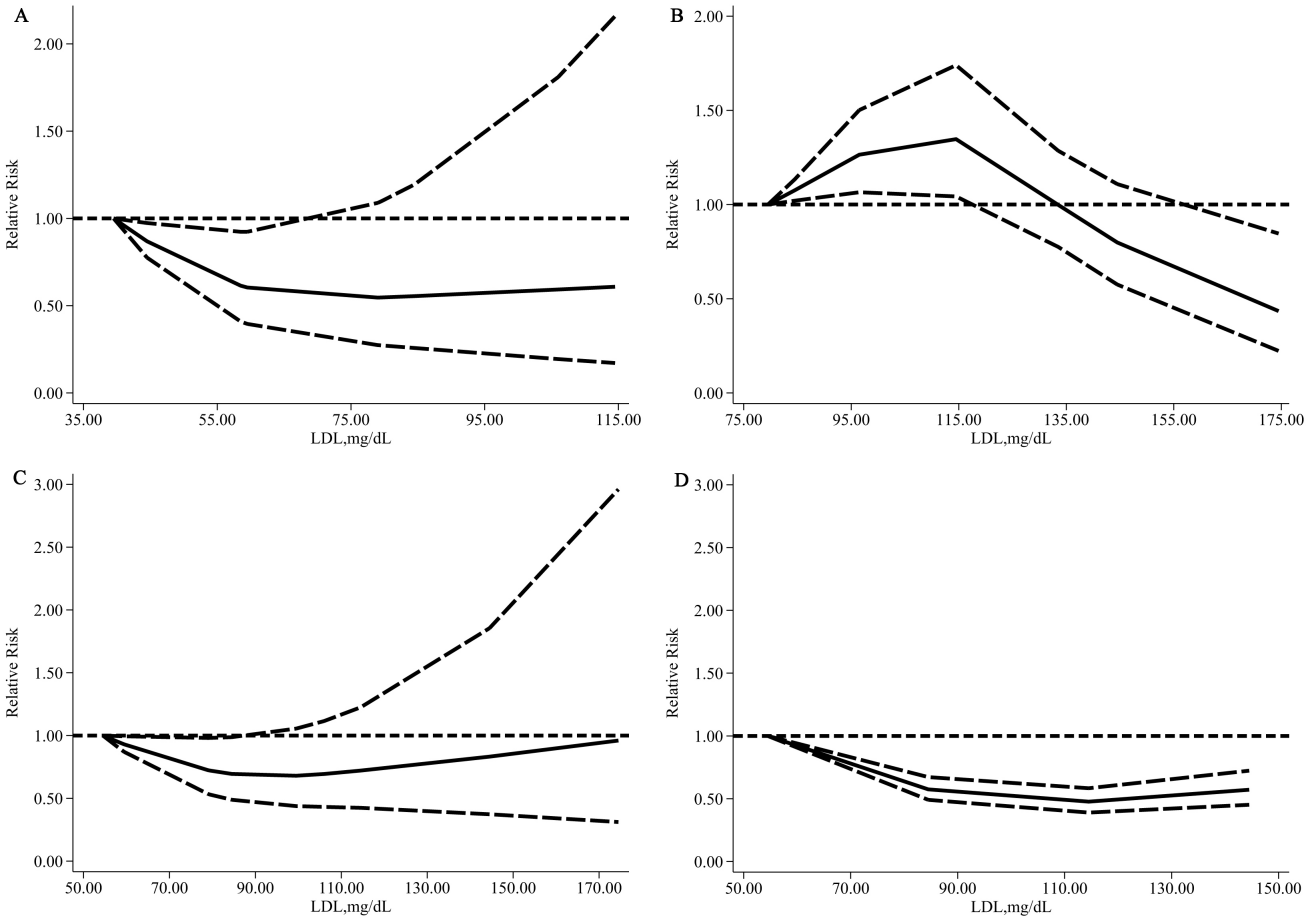
**Relative risk (solid line) with 95% CI (long dashed lines) for the association of LDL-C with the risk of HS in a hypertensive population**. (A) Hypertensive population: The reference group was LDL-C <50 mg/dL. (B) Hypertensive population: The reference group was LDL-C <70 mg/dL. (C) Hypertensive population: The reference group was LDL-C <100 mg/dL. (D) Non-hypertensive population: The reference group was LDL-C <70 mg/dL.

The non-linear trend among hypertensive populations suggested that an LDL-C level of approximately 80 mg/dL could be associated with the lowest risk of HS (Fig. 5B); the risk of HS rose as the LDL-C level increased (Fig. 5C). The risk of HS also noticeably rose when the LDL-C level was very low, below 60 mg/dL (Fig. 5A).

We detected that the lowest risk of HS among non-hypertensive populations was an LDL-C level of approximately 115 mg/dL. In comparison, the lowest risk of HS among hypertensive populations was in an LDL-C level range of 60–80 mg/dL. In addition, the degree of risk reduction of HS was greater; the effective lowering LDL-C range (95% CI of RR did not conclude 1) was wider among non-hypertensive populations than among hypertensive populations (Fig. 5B,D).

**Fig. 6.**
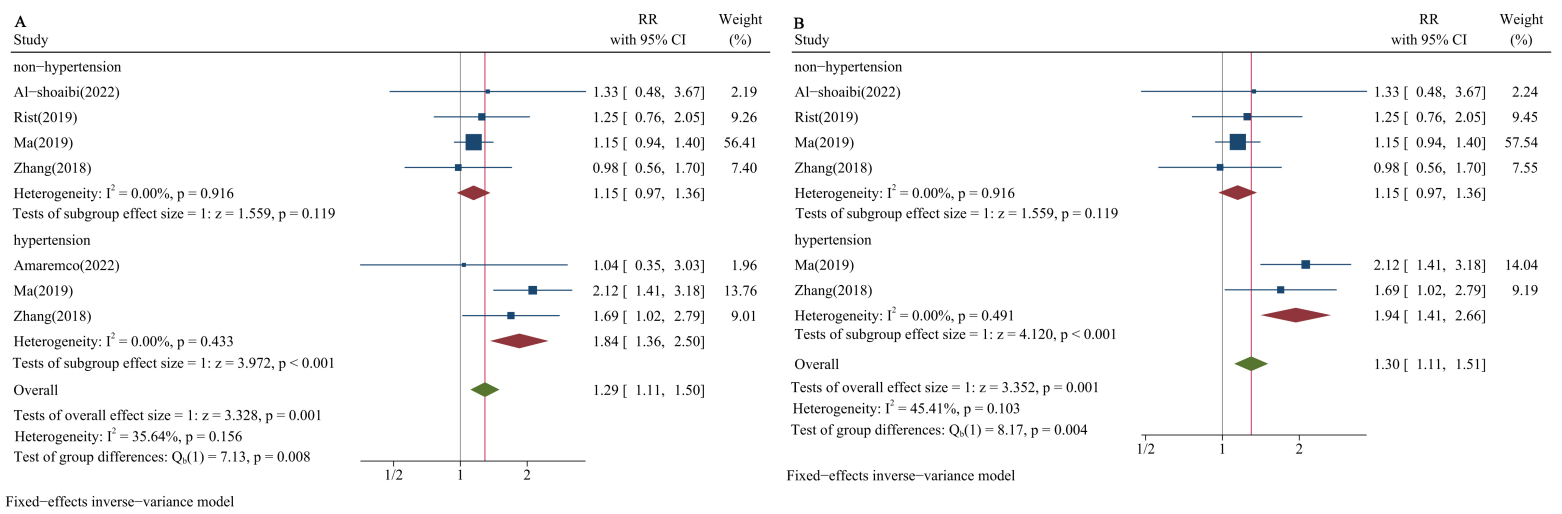
**Forest plots of LDL-C and risk of HS stratified by blood pressure status in lower versus optimal**. (A) Proportion of hypertension: 65%. (B) Proportion of hypertension: 75%.

Finally, we calculated RRs to compare the lower group adjacent to the optimal group and the optimal group among hypertensive populations (50–69 mg/dL vs. 70–99 mg/dL) and non-hypertensive populations (70–99 mg/dL vs. 100–129 mg/dL). The results showed that when the proportion of the study population with hypertension was more than 65%, the RRs were significant (RR = 1.84, 95% CI: 1.36–2.50; RR = 1.94, 95% CI: 1.41–2.66, respectively; Fig. 6A,B); the RRs were not significant among non-hypertensive populations (RR = 1.15, 95% CI: 0.97–1.36; RR = 1.15, 95% CI: 0.97–1.36, respectively; Fig. 6A,B).

## 4. Discussion

Our results showed a non-linear relationship between LDL-C and risk of HS in both hypertensive and non-hypertensive populations. However, there were still some differences in the relationship between these two populations. The non-linear trend suggested that the lowest risk of HS could be associated with an LDL-C level of approximately 80 mg/dL in the hypertensive population and 115 mg/dL in the non-hypertensive population. The reductive degree of HS risk was larger in the non-hypertensive population than in the hypertensive population when the LDL-C level was optimal. When we compared the RR of the lower group adjacent to the optimal group and the optimal group according to blood pressure status, we found that lowering LDL-C continually below the optimal group increased the risk of HS in the hypertensive population but not in the non-hypertensive population. This might mean that the population with hypertension needs more precise and rigorous control of LDL-C levels with a narrow, safe range to protect HS.

We have previously reported that low cholesterol levels may inhibit autophagy through phosphoinositide 3-kinase/protein kinase B/mammalian target of rapamycin (PI3K/Akt/mTOR) signaling pathway (PAM pathway) and induce arterial smooth muscle cell necrosis, thereby increasing the risk of ICH [39]. In this study, we found a significant relationship between LDL-C and HS. Low concentrations of LDL-C not only increase the vulnerability of the cerebrovascular wall but also increase its permeability. This can cause arterial necrosis, microaneurysm formation, changing platelet aggregation, decreasing vascular wall resistance, and eventually leading to cerebral hemorrhage [2, 40, 41]. Interestingly, recent studies using magnetic resonance imaging (MRI) have found that patients with cerebral microbleeds (CMBs) also have lower LDL-C levels [42]. Low LDL-C levels may play a role in promoting the necrosis of medial smooth muscle cells, increasing the risk of microaneurysms, which are the main pathological findings of intracranial hemorrhage events [40]. Some studies and guidelines recommend lowering LDL-C levels; some even suggest that ‘lower is better’ [31, 43, 44, 45]. However, our findings and similar results from previous studies indicated that the view of ‘lower is better’ is not always true [46, 47, 48]. At the same time, our findings suggested that the appropriate LDL-C level should be considered in different populations.

This study also found that blood pressure was the main risk factor for HS. According to the Global Burden of Disease studies, hypertension is the second leading risk factor for disability-adjusted life-years and mortality [49, 50]. Hypertensive individuals have a higher risk of stroke compared to normotensive individuals [51]. Another study has also shown that hypertension is a more significant risk factor for ICH, with a greater role than lowering LDL-C [52]. Therefore, less attention has been paid to the optimal threshold for preventing HS by reducing LDL-C in hypertensive populations. However, more than one in four people in China have hypertension [53], yet the treatment and control rate of hypertension is very low. Therefore, preventing HS in the hypertensive population should not be limited to reducing blood pressure. Our results showed that in hypertensive people with LDL-C <60 mg/dL, the risk of HS bleeding increased. A study by Asia Pacific Cohort Studies Collaboration [48] also demonstrated that a low LDL-C level was associated with an increased risk of hemorrhagic stroke in population when SBP was over 130 mmHg. In the pathological state of poor blood pressure control, a decrease in cholesterol levels can increase the fragility of cerebral vascular endothelial cells, promote the necrosis of arterial smooth muscle cells, inhibit platelet aggregation, affect the permeability fragility of red blood cells, and eventually lead to bleeding [54, 55, 56]. The high level of angiotensin (AT-II) in hypertensive patients promotes LDL-C oxidation, and oxidized LDL-C (oxLDL-C) further induces endothelial cell injury and apoptosis and inhibits platelet adhesion [57, 58]. Therefore, controlling these two factors simultaneously may have therapeutic potential. However, our results showed that the safe range of LDL-C control for preventing HS in the hypertensive population is narrower than that in the non-hypertensive population.

This study was limited because several included studies presented only baseline cholesterol data, which failed to reflect information about LDL-C level changes during follow-up. Moreover, the reference group for the LDL-C concentration and the range of LDL-C were not the same; however, we conducted subgroup analysis and dose-response analysis in different reference groups of LDL-C concentrations. Despite this limitation, our meta-analysis has several advantages: Compared with previous meta-analyses, ours only included RCTs and cohort studies to avoid recall bias, and the articles were of high quality. Our study has the advantage of longer follow-up periods. Additionally, most included studies had large sample sizes involving different general populations from countries worldwide, and the methodological quality was satisfactory.

## 5. Conclusions

Our study has important clinical and public health implications. We highlighted the protective effect of lowering LDL-C to 60–80 mg/dL in hypertensive populations and that very low LDL-C levels appear to be a risk factor for HS. Our findings can remind clinicians to exercise caution during intensive lipid-lowering therapy, particularly in hypertensive patients. This may help to improve the effectiveness of individualized patient stroke risk assessment and guide clinical decision-making. Further studies are needed to investigate the underlying pathogenesis and determine which individuals can benefit most from lowering cholesterol levels for HS. Further studies could focus on the mechanistic hypothesis that very low levels of LDL-C increase the risk of hemorrhagic stroke by reducing the integrity of blood vessel walls. Different genetic profiles modify the relationship between LDL-C levels and hemorrhagic stroke risk in hypertensive patients. We can also turn the result of this study into clinical management hypotheses, such as RCTs evaluating personalized treatment strategies and cohort studies verifying long-term outcomes.

## Availability of Data and Materials

The datasets used and/or analyzed during the current study are available from the corresponding author on reasonable request.

## Supplementary Material

Supplementary material associated with this article can be found, in the online version, at https://doi.org/10.31083/RCM36363.
