# Supplementary material for: Low-Density Lipoprotein Cholesterol Reductions of not Less Than 60 mg/dL Prevent Hemorrhagic Stroke in Hypertensive Populations: A Meta-analysis
Source: Rev Cardiovasc Med. 2025 May 27;26(5):36363. doi: 10.31083/RCM36363 (PMC12135648; doi:10.31083/RCM36363)
Supplement: Supplementary file 1 [file 2153-8174-26-5-36363-s1.zip › supplement material.docx]

Search Strategy

Pubmed

#1 "lipoproteins, ldl"[MeSH Terms] OR "lipoproteins ldl"[Title/Abstract] OR "LDL"[Title/Abstract] OR "Low Density Lipoprotein"[Title/Abstract]

#2 "cholesterol, ldl"[MeSH Terms] OR "Cholesterol,LDL"[Title/Abstract] OR "Low Density Lipoprotein Cholesterol"[Title/Abstract] OR "LDL Cholesterol" [Title/Abstract] OR "LDL Cholesteryl Linoleate"[Title/Abstract]

#3 #1 OR #2

#4 "Hemorrhagic Stroke"[MeSH Terms] OR "Hemorrhagic Stroke"[Title/Abstract] OR "HS"[Title/Abstract] OR "intracerebral hemorrhage"[Title/Abstract] OR "ICH"[Title/Abstract] OR "Subarachnoid Hemorrhagic Strokes"[Title/Abstract] OR "SAH"[Title/Abstract]

#5 #3 AND #4

#6 #5 AND humans[Filter]

Cochrane Library:

#1 MeSH descriptor:[Hemorrhagic Stroke] explode all trees

#2 ("Hemorrhagic Stroke"):ti,ab,kw OR (HS):ti,ab,kw OR ("intracerebral hemorhage"):ti,ab,kw OR (ICH):ti,ab,kw OR ("Subarachnoid Hemorrhagic"): ti,ab,kw

#3 #1 OR #2

#4 MeSH descriptor:[Cholesterol, LDL] explode all trees

#5 ("Cholesterol,LDL"):ti,ab,kw OR ("Low Density Lipoprotein Cholesterol"): ti,ab,kw OR("LDL Cholesterol"):ti,ab,kw OR ("LDL Cholesteryl Linoleate"): ti,ab,kw

#6 #4 OR #5

#7 #3 AND #6

Embase:

#1 **'low density lipoprotein'**/exp OR **'low density lipoprotein'**:ab,ti OR

#2 **'low density lipoprotein cholesterol'**/exp OR**'low density lipoprotein cholesterol'**:ab,ti OR**'ldl cholesteryl linoleate'**:ab,ti

#3 #1 OR #2

#4 **'brain hemorrhage'**/exp OR**'brain hemorrhage'**:ab,ti OR **'subarachnoid hemorrhagic strokes'**:ab,ti

#5 #3 AND #4

#6 #5 AND **'human'**/de
