# Supplementary material for: Low-Density Lipoprotein Cholesterol Reductions of not Less Than 60 mg/dL Prevent Hemorrhagic Stroke in Hypertensive Populations: A Meta-analysis
Source: Rev Cardiovasc Med. 2025 May 27;26(5):36363. doi: 10.31083/RCM36363 (PMC12135648; doi:10.31083/RCM36363)
Supplement: Supplementary file 1 [file 2153-8174-26-5-36363-s1.zip › supplemental table 1 study characteristics.docx]

Supplemental table 1 Study Characteristics

| Author, year | Study Design | Study Name | Location | Participants* | Hypertension(%) | Mean/median age (range) | Female (%) | Mean/median Follow-up duration(year) | Endpoint | First occurrence/ recurrence | Time of LDL/LDL-C measurement | LDL/LDL-C concentration | HR/RR(95%CI) | Adjusted Covariates |
| --- | --- | --- | --- | --- | --- | --- | --- | --- | --- | --- | --- | --- | --- | --- |
| Al-shoaibi,2022 | PC | The Aichi Workers’ Cohort Study | Asia (Japan) | 8996 | 23.10 | 45.6 | 1889(21.00) | 12.0 | ICH | First occurrence | Baseline (mg/dL) | <100 | 1.33(0.48-3.65) | age, sex, smoking, alcohol drinking, physical activity, body mass index, high- density lipoprotein cholesterol, triglycerides, history of diabetes and hypertension, antihypertensive medication, survey year, dyslipidemia medication. |
|  |  |  |  |  |  |  |  |  |  |  |  | 100-119 | 1.00(ref) |  |
|  |  |  |  |  |  |  |  |  |  |  |  | 120-139 | 0.39(0.11-1.31) |  |
|  |  |  |  |  |  |  |  |  |  |  |  | 140-159 | 0.46(0.11-1.83) |  |
|  |  |  |  |  |  |  |  |  |  |  |  | 160- | 0.17(0.02-1.37) |  |
| Amarenco, 2022 | RCT | TST trial | Europe (France) & Asia (South Korea) | 2860 | 65.64 | 66.7(>18 in France; ≥20 in South Korea) | 926(32.38) | 3.5 | ICH | First occurrence | On-treatment over 5 years(mg/dL) | ≥90 | 1.00(ref) | - |
|  |  |  |  |  |  |  |  |  |  |  |  | 70-89 | 0.81(0.28-2.30 |  |
|  |  |  |  |  |  |  |  |  |  |  |  | <70 | 0.84(0.31-2.25) |  |
| Rist,2019 | RCT | WHS | Americas (America) | 27937 | 25.19 | 54.71 (≥45) | 27937(100) | 19.3 | HS | First occurrence | Baseline (mg/dL) | <70 | 2.17(1.05-4.48) | age, BMI, SBP, DBP, cigarette smoking , alcohol consumption, use of lipid‐lowering drugs，diabetes, history of hypertension, and family history of CVD |
|  |  |  |  |  |  |  |  |  |  |  |  | 70-99.9 | 1.25(0.76-2.04) |  |
|  |  |  |  |  |  |  |  |  |  |  |  | 100-129.9 | 1.00(ref) |  |
|  |  |  |  |  |  |  |  |  |  |  |  | 130-159.9 | 1.14(0.72-1.80) |  |
|  |  |  |  |  |  |  |  |  |  |  |  | ≥160 | 1.53(0.92-2.52) |  |
| Jukema,2019^#^ | RCT | ODYSSEY OUTCOMES trial | Global | 18924 | 64.73 | 58.55(≥40) | 4762(25.16) | 2.8 | HS | First occurrence | achieved at month 4 (Alirocumab Treatment)  (mg/dL) | <25 | 1.00(ref) | - |
|  |  |  |  |  |  |  |  |  |  |  |  | 25-<50 | 1.36(0.23-9.14) |  |
|  |  |  |  |  |  |  |  |  |  |  |  | 50-<70 | 4.69(0.78-29.09) |  |
|  |  |  |  |  |  |  |  |  |  |  |  | ≥70 | 5.79(1.06-31.66) |  |
| Ma, 2019 | PC | the Kailuan Study | Asia (China) | 96043 | 54.13 | 51.3(≥18) | 19780(20.6) | 9.0 | ICH | First occurrence | Baseline (mg/dL(1.3mmol/L)) | <50 | 1.3(1.01-1.66) | age, sex, smoking , alcohol intake , education , physical activity , average monthly income of each family member , salt intake , updated diabetes status , use of antihypertensive, lipid-lowering agents, aspirin, and anticoagulants, updated cumulative average body mass index, triglycerides, high-density lipoprotein cholesterol, hs-CRP, and alanine aminotransferase levels, systolic blood pressure, diastolic blood pressure |
|  |  |  |  |  |  |  |  |  |  |  |  | 50-69 | 0.97(0.77-1.23) |  |
|  |  |  |  |  |  |  |  |  |  |  |  | 70-99 | 1.00(ref) |  |
|  |  |  |  |  |  |  |  |  |  |  |  | 100-129 | 1.02(0.85-1.23) |  |
|  |  |  |  |  |  |  |  |  |  |  |  | 130-159) | 0.91(0.67-1.24) |  |
|  |  |  |  |  |  |  |  |  |  |  |  | ≥160 | 1.06(0.72-1.56) |  |
| Zheng,2019 | PC | — | Asia (China) | 5097 | 100.00 | 56.3(≥35) | 2866(56.2) | 8.4 | HS | First occurrence | Baseline (mmol/L) | <2.29 | 1.00(ref) | age, sex, ethnicity, BMI, current smoking, heavy drinking, diabetes mellitus, SBP, DBP, and anti-hypertension drug treatment |
|  |  |  |  |  |  |  |  |  |  |  |  | 2.29-2.73 | 1.22(0.80-1.86) |  |
|  |  |  |  |  |  |  |  |  |  |  |  | 2.73-3.22 | 1.18(0.77-1.81) |  |
|  |  |  |  |  |  |  |  |  |  |  |  | ≥3.22 | 0.96(0.61-1.52) |  |
| Hosomi,2018 | RCT | J-STARS | Asia(Japan) | 1578 | 75.9 | 66.2(45-80) | 492(31.2) | 5.0 | ICH | First occurrence | Baseline  (mg/dL) | <100 | 1.00(ref) | body mass index, hypertension, diabetes mellitus, and statin usage |
|  |  |  |  |  |  |  |  |  |  |  |  | 100-120 | 0.17(0.04-0.74) |  |
|  |  |  |  |  |  |  |  |  |  |  |  | 120-140 | 0.30(0.09-0.96) |  |
|  |  |  |  |  |  |  |  |  |  |  |  | 140-160 | 0.11 (0.02-0.54) |  |
|  |  |  |  |  |  |  |  |  |  |  |  | ≥160 | 0.57 (0.15-2.15) |  |
| Zhang,2018 | PC | CMCS | Asia (China) | 20954 | 30.1 | 47.4(35-64) | 10163(48.5) | 16.4 | HS | First occurrence | Baseline  (mg/dL) | <70 | 1.57(1.05-2.35) | age, sex, BMI, smoking status, alcohol drinking status, family history of CVD, FBG, low HDL-C status, and lipid-lowering medication |
|  |  |  |  |  |  |  |  |  |  |  |  | 70-99 | 1.00(ref) |  |
|  |  |  |  |  |  |  |  |  |  |  |  | 100-129 | 1.34(0.97-1.85) |  |
|  |  |  |  |  |  |  |  |  |  |  |  | 130-159 | 1.10-0.74-1.64) |  |
|  |  |  |  |  |  |  |  |  |  |  |  | ≥160 | 0.83(0.46-1.49) |  |
| Giugliano,2017 | RCT | IMPROVE-IT | Europe, Americas, Asia-Pacific | 15281 | 60.7 | 63.0 | 3636(23.79) | 6.0 | HS | First occurrence | achieved at 1 month  (mg/dL) | <30 | 0.36(0.11-1.26) | Baseline Characteristics |
|  |  |  |  |  |  |  |  |  |  |  |  | 30-49 | 1.05(0.6-1.84) |  |
|  |  |  |  |  |  |  |  |  |  |  |  | 50-69 | 0.58(0.33-1.04_ |  |
|  |  |  |  |  |  |  |  |  |  |  |  | ≥70 | 1.00(ref) |  |
| Stoekenbroek,2015 | PC | EPIC-Norfolk | Europe (UN) | 21798 | 37.0 | 59(45-79) | 12222(56.0) | 12.1 | HS | First occurrence | Baseline  (mmol/L) | — | 0.75(0.46-1.27) per 1mmol/L | age, sex, smoking, BMI, diabetes, HDL-c, and systolic blood pressure. |
|  |  |  |  |  |  |  |  |  |  |  |  | <3.24 | 1.00(ref) |  |
|  |  |  |  |  |  |  |  |  |  |  |  | 3.25-3.88 | 0.79(0.47-1.33) |  |
|  |  |  |  |  |  |  |  |  |  |  |  | 3.89-4.59 | 0.76(0.45-1.28) |  |
|  |  |  |  |  |  |  |  |  |  |  |  | >4.60 | 0.72(0.43-1.22) |  |
| Wieberdink,2011 | PC | Rotterdam Study | Europe (Netherland) | 5773 | 67.29 | Rotterdam Study I(third visit):71.0; Rotterdam Study II(first visit):61.8 (≥55) | 3296（57.0） | 9.7 | ICH | First occurrence | Baseline  （mmol/L） | — | 1.07)0.78-1.46)  per 1 SD 4mmol/L | age, sex, and a propensity score of potential confounders (lipid-lowering medication use, systolic blood pressure, blood pressure-lowering medication use, diabetes mellitus, serum glucose level, serum insulin level, current cigarette smoking, body mass index, antithrombotic medication use, alcohol intake and subcohort). |
|  |  |  |  |  |  |  |  |  |  |  |  | 0.1-3.2 | 1.00(ref) |  |
|  |  |  |  |  |  |  |  |  |  |  |  | 3.2-3.7 | 1.03(0.44-2.41) |  |
|  |  |  |  |  |  |  |  |  |  |  |  | 3.7-4.3 | 1.47(0.65-3.33) |  |
|  |  |  |  |  |  |  |  |  |  |  |  | 4.3-7.9 | 0.98(0.39-2.50) |  |
| Imamura,2009 | PC | Hisayama Study | Asia (Japan) | 2351 | 43.35 | 57.25(≥40) | 1360(57.85) | 19.0 | HS | First occurrence | Baseline  (mmol/L) | ≤2.65 | 1.00(ref) | age, sex, HDL cholesterol, triglycerides, systolic BP, ECG abnormalities, fasting blood glucose, BMI, current drinking, current smoking, and regular exercise. |
|  |  |  |  |  |  |  |  |  |  |  |  | 2.66-3.24 | 0.71(0.34-1.47) |  |
|  |  |  |  |  |  |  |  |  |  |  |  | 3.25-3.88 | 1.41(0.75-2.65) |  |
|  |  |  |  |  |  |  |  |  |  |  |  | ≥3.89 | 1.01(0.50-2.05) |  |
| Goldstein,2008 | RCT | SPARCL | Americas; Europe; Australia | 4731 | 61.89 | 62.75(≥18) | 1916(40.5) | 4.9 | HS | recurrence | Baseline  (mg/dL) | <52 | 1.00(ref) | age, region, qualifying event, days since qualifying event, sex,SBP, DBP, LDL-C, and HDL-C |
|  |  |  |  |  |  |  |  |  |  |  |  | 52-65 | 1.26(0.60-2.64) |  |
|  |  |  |  |  |  |  |  |  |  |  |  | 66-92 | 0.97(0.44-2.17) |  |
|  |  |  |  |  |  |  |  |  |  |  |  | ≥93 | 1.37(0.63-2.98) |  |
| Sturgeon,2007 | PC | ARIC&CHS | Americas (America) | ARIC:15463  CHS:5888 | ARIC:35.4  CHS:65.9 | ARIC:54.2(45-64)  CHS:72.8(≥65) | ARIC:8717(55.2)  CHS:3391(57.6) | ARIC:7.2  CHS:7.5 | ICH | First occurrence | Baseline  (mg/dL) | — | 0.86(0.59-1.27)  per 10mg/dL | age |
|  |  |  |  |  |  |  |  |  |  |  |  | 4.0-109.6 | 1.00(ref) |  |
|  |  |  |  |  |  |  |  |  |  |  |  | 109.7-133.0 | 0.88(0.56-1.39) |  |
|  |  |  |  |  |  |  |  |  |  |  |  | 133.0-158.8 | 0.85(0.53-1.35) |  |
|  |  |  |  |  |  |  |  |  |  |  |  | 158.8-504.6 | 0.50(0.29-0.87) |  |
| Waters,2006^#^ | RCT | Treating to New Targets Study | Americas (America) | 10001 | 54.12 | 61.1(35-75) | 1902(19.01) | 4.9 | HS | First occurrence | 3 months after randomation (mg/dL) | <64 | 1.00(ref) | - |
|  |  |  |  |  |  |  |  |  |  |  |  | 64-77 | 0.79(0.24-2.60) |  |
|  |  |  |  |  |  |  |  |  |  |  |  | 77-90 | 0.92(0.30-2.87) |  |
|  |  |  |  |  |  |  |  |  |  |  |  | 90-106 | 1.36(0.48-3.82) |  |
|  |  |  |  |  |  |  |  |  |  |  |  | ≥106 | 1.08(0.36-3.22) |  |
| Nakaya,2005 | PC | J-LIT | Asia (Japan) | 41088 | 45.6 | 57.7(male:35-70; post-menopausal female:<70) | 28104(68.4) | 5.39 | Cerebral hemorrhage (Intracranial) | First occurrence | Baseline  (mg/dL) | ＜120 | 1.00(ref) | gender and age at baseline, hypertension, diabetes mellitus, and smoking habit. |
|  |  |  |  |  |  |  |  |  |  |  |  | 120-139 | 1.41(0.88-2.26) |  |
|  |  |  |  |  |  |  |  |  |  |  |  | 140-159 | 0.82(0.44-1.51) |  |
|  |  |  |  |  |  |  |  |  |  |  |  | 160-179 | 0.95(0.42-2.16) |  |
|  |  |  |  |  |  |  |  |  |  |  |  | ≥180 | 1.51(0.62-3.63) |  |

*: available for analysis

^#^: RR was calculated according to the number of sample and event in the study by logistic regression.

Abbreviations: RCT: Randomized Control Trail; PC: Prospective Cohort; NCC: Nested Case Control; FOURIER-OLE: Further Cardiovascular Outcomes Research With PCSK9 Inhibition in Subjects With Elevated Risk Open-Label Extension; TST trial: Treat Stroke to Target; CHNS: The China Health and Nutrition Survey; CKB: China Kadoorie Biobank; WHS: Women’s Health Study; ODYSSEY OUTCOMES trial: Evaluation of Cardiovascular Outcomes After an Acute Coronary Syndrome During Treatment With Alirocumab; CMCS: China Multi-Provincial Cohort Study; J-STARS: Japan Statin Treatment Against Recurrent Stroke; IMPROVE-IT: The Improved Reduction of Outcomes: Vytorin Efficacy International Trial; REGARDS: Reasons for Geographic And Racial Differences in Stroke study; EPIC: The European Prospective Investigation of Cancer; SPARCL: The Stroke Prevention by Aggressive Reduction in Cholesterol Levels Trail; AMORIS: Apolipoprotein Mortality Risk study; ARIC: Atherosclerosis Risk in Communities Study; CHS: Cardiovascular Health Study; J-LIT: Japan Lipid Intervention Trial; HS: Hemorrhagic Stroke; ICH: intracerebral hemorrhage; SAH: Subarachnoid Hemorrhagic Strokes.
