# Supplementary material for: Low-Density Lipoprotein Cholesterol Reductions of not Less Than 60 mg/dL Prevent Hemorrhagic Stroke in Hypertensive Populations: A Meta-analysis
Source: Rev Cardiovasc Med. 2025 May 27;26(5):36363. doi: 10.31083/RCM36363 (PMC12135648; doi:10.31083/RCM36363)
Supplement: Supplementary file 1 [file 2153-8174-26-5-36363-s1.zip › supplemental table 2 NOS.docx]

Supplemental table 2 quality assessment of prospective cohort studies by NOS

| Author，year | Selection | | | |  | Comparability of cohorts | |  | Outcome | | | Total score |
| --- | --- | --- | --- | --- | --- | --- | --- | --- | --- | --- | --- | --- |
|  | Representativeness of exposed cohort | Representativeness of non-exposed cohort | Ascertainment of exposure | Outcome not present at beginning of study |  | Control for age | Control for other confounding factors |  | Assessment of outcome | Was follow-up long enough? | Adequacy of follow-up |  |
| Al-shoaibi,2022 | 0 | 1 | 1 | 1 |  | 1 | 1 |  | 1 | 1 | 1 | 8 |
| Ma, 2019 | 1 | 1 | 1 | 1 |  | 1 | 1 |  | 1 | 1 | 1 | 9 |
| Zheng,2019 | 1 | 1 | 1 | 1 |  | 1 | 1 |  | 1 | 1 | 1 | 9 |
| Zhang,2018 | 1 | 1 | 1 | 1 |  | 1 | 1 |  | 1 | 1 | 1 | 9 |
| Stoekenbroek,2015 | 1 | 1 | 1 | 1 |  | 1 | 1 |  | 1 | 1 | 0 | 8 |
| Wieberdink,2011 | 1 | 1 | 1 | 1 |  | 1 | 1 |  | 1 | 1 | 1 | 9 |
| Imamura,2009 | 1 | 1 | 1 | 1 |  | 1 | 1 |  | 1 | 1 | 1 | 9 |
| Sturgeon,2007 | 1 | 1 | 1 | 1 |  | 1 | 0 |  | 1 | 1 | 0 | 7 |
| Nakaya,2005 | 1 | 1 | 1 | 1 |  | 1 | 1 |  | 1 | 1 | 1 | 9 |

Studies with more than six scores were regarded as high quality.
