# Supplementary figures and images for: Low-Density Lipoprotein Cholesterol Reductions of not Less Than 60 mg/dL Prevent Hemorrhagic Stroke in Hypertensive Populations: A Meta-analysis
Source: Rev Cardiovasc Med. 2025 May 27;26(5):36363. doi: 10.31083/RCM36363 (PMC12135648; doi:10.31083/RCM36363)

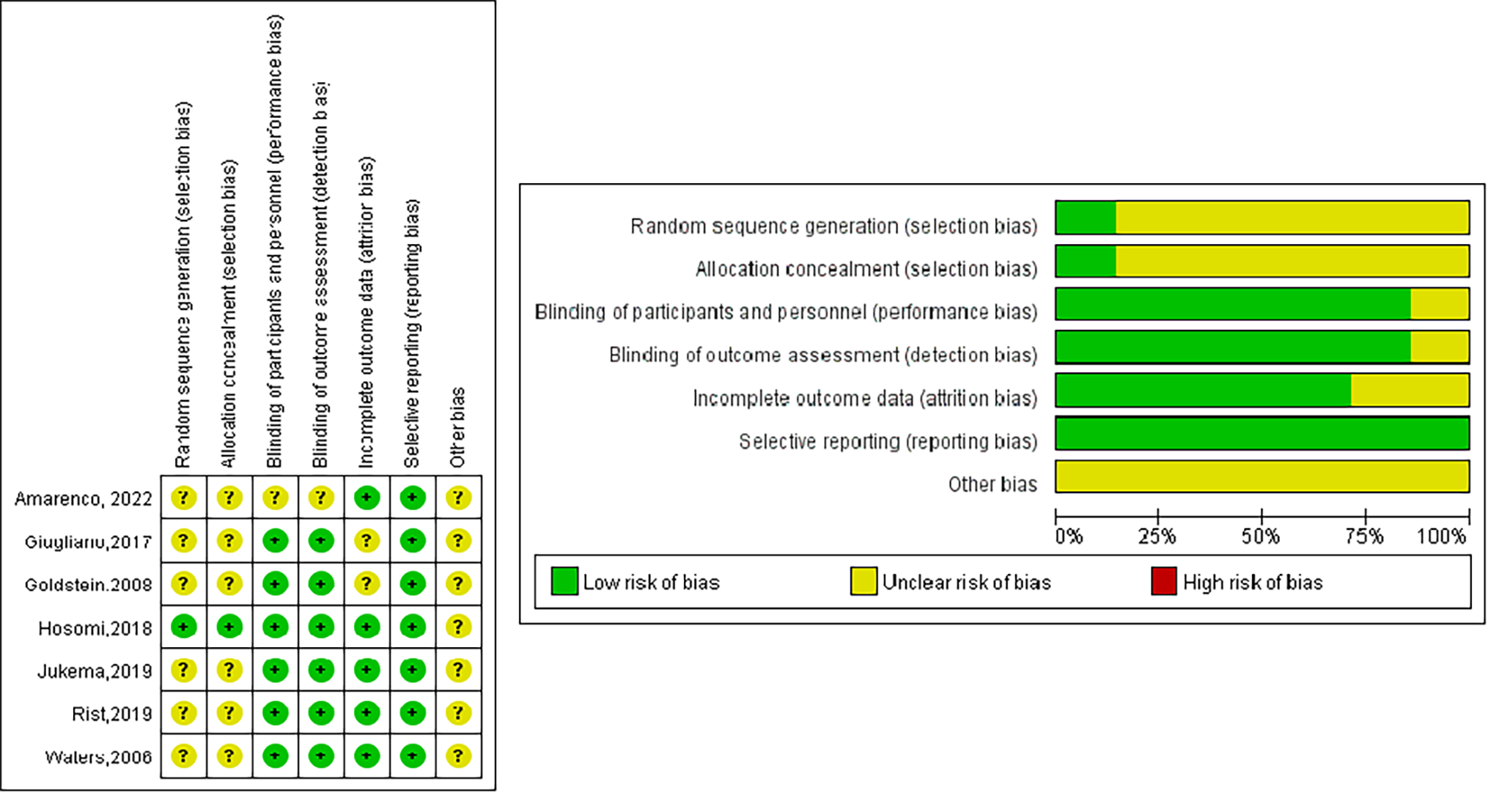

Supplement: Supplementary file 1 [file 2153-8174-26-5-36363-s1.zip › supplemental figure 1 Risk of bias.png]

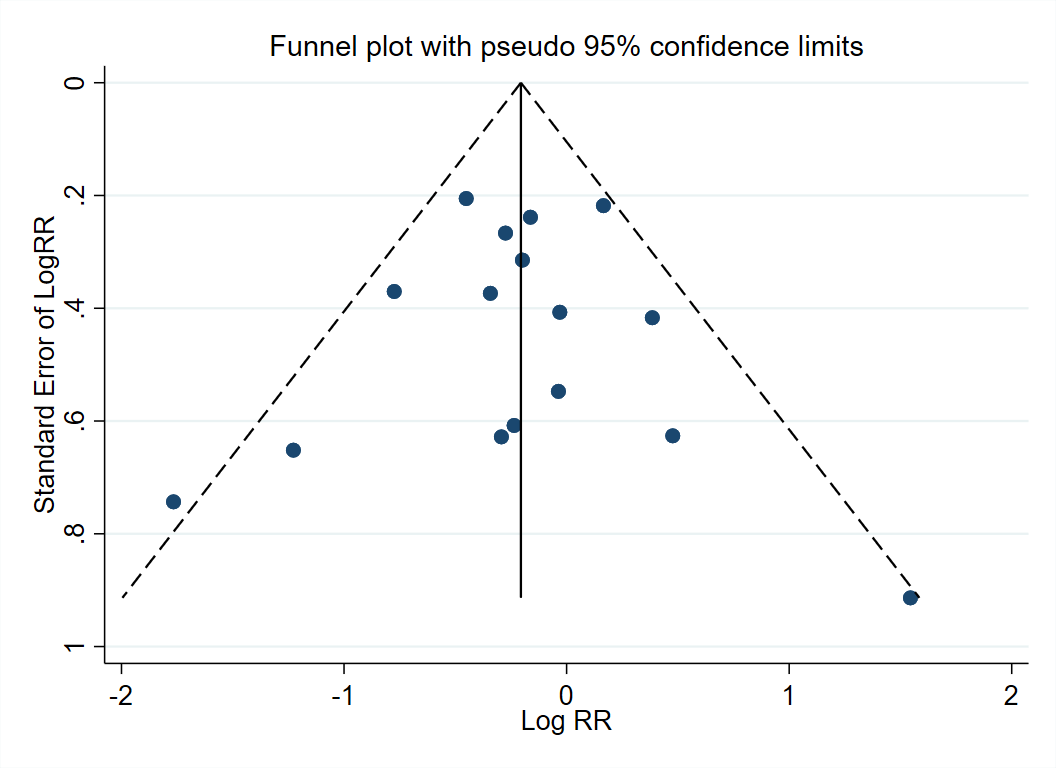

Supplement: Supplementary file 1 [file 2153-8174-26-5-36363-s1.zip › supplemental figure 2 funnel plot.png]

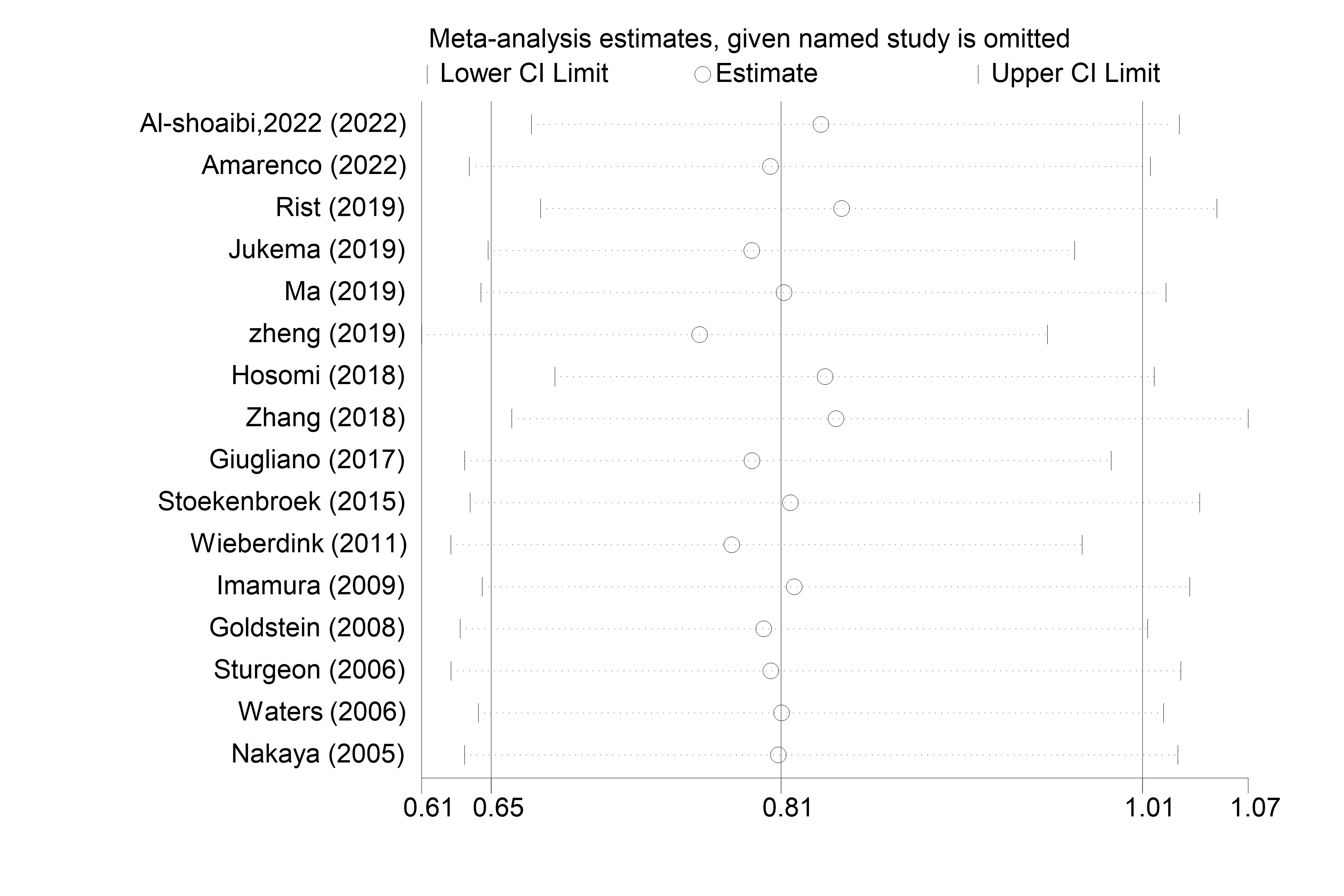

Supplement: Supplementary file 1 [file 2153-8174-26-5-36363-s1.zip › supplemental figure 3 sensitivity analysis.png]
